# Economic Evaluation of Population-Based *BRCA1* and *BRCA2* Testing in Canada

**DOI:** 10.1001/jamanetworkopen.2024.32725

**Published:** 2024-09-12

**Authors:** Li Sun, Xia Wei, Caitlin T. Fierheller, Lesa Dawson, Samuel Oxley, Ashwin Kalra, Jacqueline Sia, Fabio Feldman, Stuart Peacock, Kasmintan A. Schrader, Rosa Legood, Janice S. Kwon, Ranjit Manchanda

**Affiliations:** 1 Department of Health Services Research and Policy, London School of Hygiene & Tropical Medicine, London, United Kingdom; 2 Centre for Cancer Screening, Prevention and Early Diagnosis, Wolfson Institute of Population Health, Queen Mary University of London, London, United Kingdom; 3 Department of Obstetrics and Gynecology, University of British Columbia, Vancouver, Canada; 4 Department of Gynaecological Oncology, Barts Health NHS Trust, Royal London Hospital, London, United Kingdom; 5 Prevention, Screening, Hereditary Cancer Program and Quality, Safety & Accreditation, BC Cancer Agency, Vancouver, Canada; 6 Faculty of Health Sciences, Simon Fraser University, Burnaby, Canada; 7 Canadian Centre for Applied Research in Cancer Control, Vancouver, Canada; 8 Hereditary Cancer Program, BC Cancer Agency, Vancouver, Canada; 9 Department of Medical Genetics, University of British Columbia, Vancouver, Canada; 10 MRC Clinical Trials Unit at UCL, Institute of Clinical Trials & Methodology, Faculty of Population Health Sciences, University College London, London, United Kingdom

## Abstract

**Importance:**

Population-based *BRCA* testing can identify many more *BRCA* carriers who will be missed by the current practice of *BRCA* testing based on family history (FH) and clinical criteria. These carriers can benefit from screening and prevention, potentially preventing many more breast and ovarian cancers and deaths than the current practice.

**Objective:**

To estimate the incremental lifetime health outcomes, costs, and cost-effectiveness associated with population-based *BRCA* testing compared with FH-based testing in Canada.

**Design, Setting, and Participants:**

For this economic evaluation, a Markov model was developed to compare the lifetime costs and outcomes of *BRCA1/BRCA2* testing for all general population women aged 30 years compared with FH-based testing. *BRCA* carriers are offered risk-reducing salpingo-oophorectomy to reduce their ovarian cancer risk and magnetic resonance imaging (MRI) and mammography screening, medical prevention, and risk-reducing mastectomy to reduce their breast cancer risk. The analyses were conducted from both payer and societal perspectives. This study was conducted from October 1, 2022, to February 20, 2024.

**Main Outcomes and Measures:**

Outcomes of interest were ovarian cancer, breast cancer, additional heart disease deaths, and incremental cost-effectiveness ratio ICER per quality-adjusted life-year (QALY). One-way and probabilistic-sensitivity-analyses (PSA) were undertaken to explore the uncertainty.

**Results:**

In the simulated cohort of 1 000 000 women aged 30 years in Canada, the base case ICERs of population-based *BRCA* testing were CAD $32 276 (US $23 402.84) per QALY from the payer perspective or CAD $16 416 (US $11 903.00) per QALY from the societal perspective compared with FH-based testing, well below the established Canadian cost-effectiveness thresholds. Population testing remained cost-effective for ages 40 to 60 years but not at age 70 years. The results were robust for multiple scenarios, 1-way sensitivity, and PSA. More than 99% of simulations from payer and societal perspectives were cost-effective on PSA (5000 simulations) at the CAD $50 000 (US $36 254.25) per QALY willingness-to-pay threshold. Population-based *BRCA* testing could potentially prevent an additional 2555 breast cancers and 485 ovarian cancers in the Canadian population, corresponding to averting 196 breast cancer deaths and 163 ovarian cancer deaths per 1 000 000 population.

**Conclusions and Relevance:**

In this economic evaluation, population-based *BRCA* testing was cost-effective compared with FH-based testing in Canada from payer and societal perspectives. These findings suggest that changing the genetic testing paradigm to population-based testing could prevent thousands of breast and ovarian cancers.

## Key Points

**Question:**

Is population-based *BRCA* testing cost-effective in Canada?

**Findings:**

In this economic evaluation using 1 000 000 simulated women in a Markov model, population-based *BRCA* genetic testing was cost-effective compared with family history–based testing.

**Meaning:**

These findings suggest that many more breast and ovarian cancer cases and deaths could be prevented using a population-based *BRCA* genetic testing strategy, calling for implementation studies for this approach.

## Teaser

This economic evaluation estimates the incremental lifetime health outcomes, costs, and cost-effectiveness associated with population-based *BRCA* testing compared with family history–based testing in a simulated cohort of women in Canada.

## Introduction

Carriers of pathogenic variants (PV) in *BRCA1* (OMIM 113705) and *BRCA2* (OMIM 600185) are at increased risk of breast cancer (BC) and ovarian cancer (OC), with absolute risks of 61% to 72% and 17% to 48%, respectively, up to age 80 years.^1,2^ Current Canadian national and international guidelines recommend women undertake *BRCA1/BRCA2* genetic testing if they fulfil established clinical or family history (FH) criteria. These criteria aim to identify individuals with a 5% to 10% probability of carrying *BRCA1*/*BRCA2* PVs,^3,4,5^ but this misses approximately 50% of PV carriers in individuals with cancer^6,7,8^ and much higher proportions with population ascertainment.^9^ Implementation of clinical criteria and FH–based testing is dependent on cancer diagnoses, awareness of importance and accuracy of cancer FH, discussion between and within families, and timely referrals to genetic testing. There is underutilization and restricted access to genetic testing services across health systems due to limited awareness, complex structures of current pathways, and limited numbers of trained counsellors to provide genetic counselling.^10,11,12^ Only 20% to 40% of eligible individuals get referred for genetic testing,^11^ with rates for cascade testing and testing among racial and ethnic minority groups (ie, individuals of non-White European ethnicity, background, or ancestry) being worse.^13,14^ Resultantly, approximately 97% of PV carriers remain undetected despite *BRCA* testing having been available for approximately 30 years.^12^ This high rate of undetected carriers translates into thousands of new BC and OC diagnoses every year. *BRCA* testing is considered a Tier 1 genomic application, as it has a significant potential for positive impact on public health based on existing evidence-based guidelines and recommendations.^15^ Effective clinical management strategies are available for unaffected *BRCA* carriers.^16^ High-risk *BRCA* PV carriers can opt for risk-reducing salpingo-oophorectomy (RRSO) to reduce their OC risk^17^; magnetic resonance imaging (MRI) and mammography screening, medical prevention,^18,19^ and/or risk-reducing mastectomy (RRM)^20^ to reduce the BC risk; and/or make reproductive choices, including preimplantation genetic testing.

The limitations of clinical criteria and FH–based genetic testing can be overcome by a population-based approach offering genetic testing to individuals regardless of FH, identifying more PV carriers who can benefit from precision prevention. Population-based *BRCA* testing is the first exemplar for application of population genomics for disease prevention. Large-scale studies in Canada, the UK, Israel, the US, and Australia have evaluated this in Jewish populations (with 5-fold higher *BRCA1/BRCA2* prevalence compared with the general population, at approximately 1 carrier per 40 people vs 1 carrier per 200 people).^21,22^ Population-based *BRCA* testing in Jewish populations was implemented in Israel in 2022 and the UK in 2024.^22,23,24^ It is unknown how this could translate to the general population; Jewish population data and experiences cannot be directly extrapolated to the general Canadian population.

A recent British Columbia Gynecologic Cancer Initiative summit involving national and international experts, patient groups, and health system stakeholders, highlighted steps toward a population-based *BRCA* testing strategy for Canada. They recommended health economic assessment as a key research priority.^25^ We aim to estimate incremental lifetime effects, costs, and cost-effectiveness of population-based *BRCA1/BRCA2* genetic testing compared with clinical criteria and FH–based genetic testing in Canada.

## Methods

This economic evaluation received ethics approval from the Institute of Child Health and Great Ormond Street Hospital Research Ethics Committee with a waiver of informed consent because the study did not involve human participants. This study followed the Consolidated Health Economic Evaluation Reporting Standards (CHEERS) reporting guideline.

### Model

**Figure 1.**
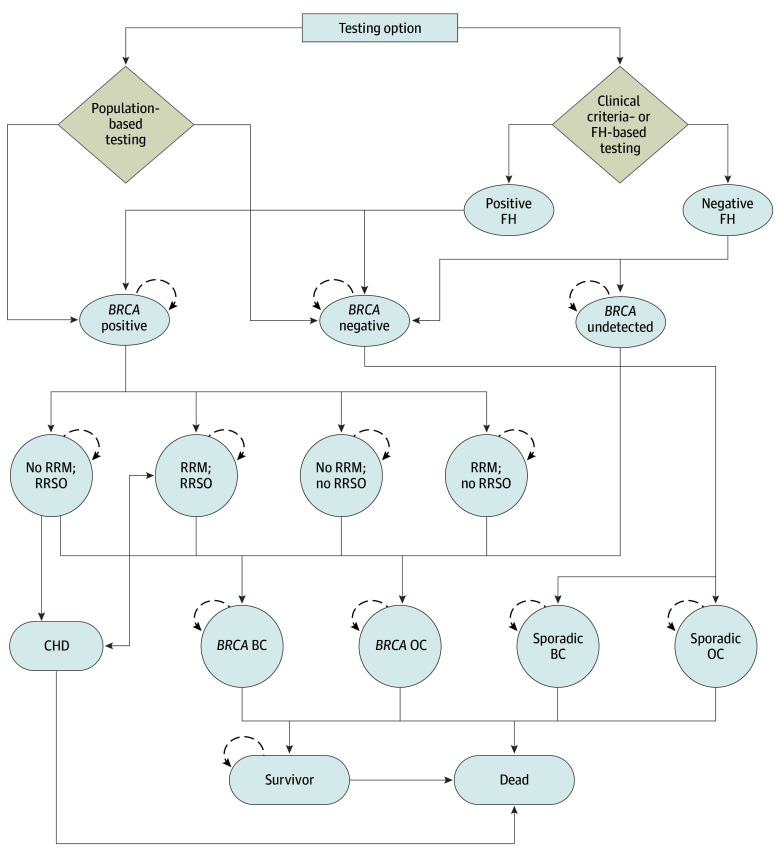
Markov Model Structure Progression through the model is dependent on the probabilities provided in Table 1. In the population testing group, all women aged 30 years are offered *BRCA1/BRCA2* testing and get classified as *BRCA* positive (ie, pathogenic variant carriers) or *BRCA* negative. A small proportion may also have variants of uncertain significance. All individuals receive pretest counselling, and posttest counselling is provided to *BRCA* pathogenic variant carriers and those with variants of uncertain significance. Identified carriers of *BRCA* pathogenic variants are offered options of risk-reducing mastectomy (RRM) and risk-reducing salpingo-oophorectomy (RRSO). In the clinical criteria and family history (FH)–based testing group, only women with FH that fulfils current clinical criteria (based on current guidelines) undergo *BRCA1/BRCA2* genetic testing and get classified as *BRCA* positive or *BRCA* negative. A small proportion may also have variants of unknown significance. All individuals receive pretest counselling, and posttest counselling is provided to carriers of *BRCA* pathogenic variants and those with variants of uncertain significance. Women with a negative FH are either *BRCA* negative or have an undetected *BRCA* pathogenic variant. Options of RRM and RRSO and disease progression for identified carriers of *BRCA* pathogenic variants and disease progression for *BRCA* negative women are the same as those in the population testing group. A detailed description of the model is given in eMethods 1 in Supplement 1. BC indicates breast cancer; CHD, coronary heart disease; OC, ovarian cancer. Arrows with dashed lines indicate that women can stay in the same health state for each cycle.

We developed a Markov model (Figure 1; eMethods 1 in Supplement 1) using TreeAge Pro software version 2018 (TreeAge software) to evaluate the lifetime costs, outcomes, and cost-effectiveness of population-based *BRCA1/BRCA2* genetic testing among women aged 30 years compared with clinical criteria and FH–based testing.^3,17,18,20,26,27,28,29,30,31,32,33,34,35,36,37,38,39,40,41,42,43,44^ Clinical- and FH-based criteria include patients with a personal history of epithelial OC, triple-negative BC, and/or FH of BC or OC in at least 1 first-degree relative. For our analysis, all women aged 30 years in the population-based testing group and only those fulfilling FH-based criteria in the FH-based testing group were offered *BRCA1/BRCA2* testing. Identified carriers of *BRCA1/BRCA2* PV were offered RRSO,^17,26^ MRI or mammography screening, medical prevention,^45^ and/or RRM.^3,20^ Women with *BRCA*-negative or undetected results were assumed to receive mammography every 2 years from age 50 to 74 years, as in general population,^46^ while women with *BRCA*-positive results receive enhanced screening of annual mammogram from age 40 to 69 years and annual MRI from age 30 to 49 years.^47^ The model incorporates the possibility of variant of uncertain significance (VUS) results,^48^ its potential reclassification to PV in the future, and associated costs and health outcomes. Given the excellent characterization of *BRCA1/2* genes and high-quality sequencing now available, our base case assumes more than 99.9% sensitivity and specificity. However, we explore a lower sensitivity of 97%^49^ in a scenario analysis. Among premenopausal women undergoing RRSO, 80% were assumed to receive hormone replacement therapy (HRT) until age 51 years, the mean age of menopause in Canada.^50^ The model incorporates increased risk of coronary heart disease (CHD) for individuals who undergo RRSO and do not use HRT.^28,51^ Model outcomes included BC, OC, and excess deaths due to CHD. The analysis was conducted from health care payer and societal perspectives. In line with the Guidelines for the Economic-Evaluation of Health Technologies by Canadian Agency for Drugs and Technologies in Health (CADTH),^52^ costs and health outcomes were discounted at 1.5%.

### Probabilities

Probabilities of the pathways in the model are shown in Table 1 and eTable 1 in Supplement 1.^3,17,18,20,26,27,28,29,30,31,32,33,34,35,36,37,38,39,40,41,42,43,44^ Age-specific incidences of BC and OC among general population women were obtained from Canadian Cancer Statistics 2021.^54^ Synchronous BC and OC is rare, so probability was presumed near zero. Age-specific BC and OC incidence for carriers of *BRCA1/BRCA2* was obtained from the literature^2^ for age 30 to 80 years, and incidence after age 80 years was assumed to be same as that of age 71 to 80 years.

**Table 1.** Model Parameters

Parameters	Estimate	Measure of variance	Source
Probabilities, % (95% CI)			
*BRCA1/2* PV prevalence in general population	0.0067	(0.0059-0.0077)	Jervi et al,^34^ 2015
Probability that carriers will undergo RRM	0.359	(0.287-0.431)	Metcalfe et al,^27^ 2019
Reduction in OC risk from RRSO (range)	0.96	(0.80-0.96)	Finch et al,^26^ 2006; Rebbeck et al,^17^ 2009
Probability of having a strong FH fulfilling genetic testing criteria	0.0098	(0.0047-0.0179)	ABCFS
*BRCA1/2* PV prevalence in individuals with FH	0.1	NA	NICE,^3^ 2023
*BRCA1/2* PV prevalence in individuals without FH	0.0058	(0.0051-0.0068)	Jervis et al,^34^ 2015; ABCFS
Reduction in BC risk from RRM without RRSO in *BRCA1/2* PV carriers (range)	0.91	(0.62-0.98)	Rebbeck et al,^20^ 2004
Probability that carriers will undergo RRSO	0.628	(0.502-0.754)	Hanley et al,^35^ 2019
HR in BC risk from RRSO alone	0.49	(0.37,0.65)	Rebbeck et al,^17^ 2009
Reduction in risk of BC from RRM with RRSO	0.95	(0.78-0.99)	Rebbeck et al,^20^ 2004
Excess CHD risk	0.0072	(0.0068-0.0076)	Parker et al,^28^ 2013
Fatal CHD risk	0.0303	(0.011-0.043)	Parker et al,^28^ 2013
Compliance with HRT	0.8	(0.76-0.83)	Read et al,^36^ 2010
HR of BC risk from BC chemoprevention	0.71	(0.6-0.83)	Cuzick et al,^18^ 2015
Uptake of BC chemoprevention	0.086	(0.069-0.103)	Metcalfe et al,^27^ 2019
Costs, CAD$ (US$)^a^			
Cost of genetic testing	220 (160.60)	±30%	Narod et al,^37^ 2021
Cost of genetic counselling	167 (121.91)		Unit cost
Cost of RRSO	4901 (3577.73)		Ministry of Health;^29^ Canadian Institute for Health Information^30^
Cost of OC diagnosis and treatment	21 800 (15 914.00)		Ministry of Health,^29^ 2021; Canadian Institute for Health Information,^30^ 2021; Oliveira et al,^31^ 2017; Sask Cancer Agency,^38^ 2017
Annual cost of OC in years 1-2	7300 (5329)		
Annual cost of OC in years 3-5	7010 (5117.30)		
Terminal care cost with OC	52 697 (38 468.81)		Oliveira et al,^31^ 2017
Cost of RRM	12 330 (9000.90)		Ministry of Health,^29^ 2021; Canadian Institute for Health Information,^30^ 2021
Annual cost of HRT	680 (496.40)		Ministry of Health,^29^ 2021
Cost of mammography	144 (105.12)		Ministry of Health,^29^ 2021
Cost of MRI	130 (94.90)		Ministry of Health,^29^ 2021
Cost of BC diagnosis and treatment in general population	33 155 (24 203.15)		Ministry of Health,^29^ 2021; Canadian Institute for Health Information,^30^ 2021; Sask Cancer Agency,^38^ 2017; Statistics Canada,^39^ 2018; Wapnir et al,^40^ 2006; Anderson et al,^41^ 2009
Annual cost of BC in general population	1414 (1032.22)		
Cost of BC diagnosis and treatment in *BRCA1/2* PV carriers	33 155 (24 203.15)		
Annual cost of BC in *BRCA1/2* PV carriers	1284 (937.32)		
Terminal care cost with BC	43 638 (31 855.74)		De Oliveira et al,^31^ 2017
Cost of fatal CHD	4839 (3532.47)		Nova Scotia Health system et al,^42^ 2023
Annual cost of excess CHD	175 (127.75)		Tran et al,^43^ 2021
Annual cost of chemoprevention	293 (213.89)		Sask Cancer Agency,^38^ 2017
Utility scores, mean (SD)			
RRM	0.88	(0.22)	Grann et al,^33^ 2010
RRSO	0.95	(0.10)	Grann et al,^33^ 2010
BC			
Early BC	0.71	±10%	NICE,^32^ 2009
Advanced BC	0.65		
Recurrent BC	0.45		
Remittent BC	0.81		
Terminal BC	0.16		
OC			
Early OC	0.81	±10%	Havrilesky et al,^53^ 2009
Advanced OC	0.55		
Recurrent OC	0.61		
Remittent OC	0.83		
Terminal OC	0.16		

Abbreviations: ABCFS, Australia Breast Cancer Family Study; BC, breast cancer; CHD, coronary heart disease; FH, family history; HR, hazard ratio; HRT, hormone replacement therapy; MRI, magnetic resonance imaging; NICE, National Institute for Health and Care Excellence; OC, ovarian cancer; PV, pathogenic variant; RRM, risk-reducing mastectomy; RRSO, risk-reducing salpingo-oophorectomy.

^a^ Cost of ovarian cancer treatment is common for the general population and *BRCA* carriers, and the cost of BC treatment is provided separately for *BRCA1/2* PV carriers and noncarriers.

### Costs

Costs are reported in 2022 Canadian dollars (with conversion to 2024 US dollars). We collected primary data on relevant direct medical costs from the Medical Services Commission Payment Schedule in Canada,^29^ the Canadian Institute for Health Information Patient Cost Estimator,^30^ and published literature^31^ (Table 1; eTable 2 in Supplement 1). We adopted internationally available *BRCA* testing costs and explored the impact of change in testing costs on base case results in the sensitivity analyses. We categorized costs due to productivity loss (eMethods 2 in Supplement 1), including temporary disability from short-term work absences following diagnosis, permanent disability from reduced working hours following return to work or workforce departure, and premature mortality from death before retirement.^55^

### Life-Years

The model simulation started at age 30 years and cycled annually until age 83 (female life expectancy in Canada).^56^ The lifetime table from Canada was used to model the lifetime health outcomes, obtained from Statistics Canada.^56^ The median ages for RRM and RRSO in unaffected carriers of *BRCA1/BRCA2* PV were assumed to be 37 and 40 years, respectively,^57^ and these were varied in the scenario analyses. When model simulation began at ages 40 to 70, RRM and RRSO occurred in the next cycle, as these are older than the median ages for either surgery. BC and OC survival were modeled using 5-year survival data from Canadian Cancer Statistics 2022^54^ and published literature^58,59^ (eMethods 3 in Supplement 1). To our knowledge, no significant long-term survival differences between hereditary (*BRCA1/BRCA2*) and sporadic BC and OC have been found.^60,61,62^ After 5 years, women diagnosed with BC or OC were assumed to have a probability of background all-cause mortality.

### Quality-Adjusted Life-Years

Quality-adjusted life years (QALYs) is the preferred outcome measure recommended by CADTH in economic evaluation.^52^ Utility scores are multiplied by life-years to obtain QALYs. Utility score is an indication of individual preferences for specific health states, where 1 indicates perfect health and 0 indicates death, reflecting an adjustment for quality of life. The utility score for early BC is 0.71; advanced BC, 0.65; recurrent BC, 0.45; remission, 0.81; and end-stage BC, 0.16,^32^ while the utility score for early OC is 0.81; advanced OC, 0.55; recurrent OC, 0.61; remission, 0.83; and end-stage OC, 0.16.^53^ Additionally, utility scores for RRM (mean [SD], 0.88 [0.22]) and RRSO (mean [SD], 0.95 [0.10]) were incorporated.^33^

### Statistical Analysis

This study was conducted from October 1, 2022, to February 20, 2024. The incremental cost-effectiveness ratio (ICER) was calculated by dividing the difference in cost by the difference in health outcomes between the 2 strategies (population-based vs FH-based testing). ICERs were compared with the willingness-to-pay (WTP) thresholds of CAD $50 000 (US $36 254.25) per QALY and CAD $100 000 (US $72 508.50) per QALY, which are conventionally used in Canada. The population impact was estimated by calculating the reduced incidence of and deaths from BC and OC over a lifetime horizon by offering population-based *BRCA1/BRCA2* testing to women aged 30 years.

We explored several scenario analyses: (1) genetic testing offered at older ages of 40 years, 50 years, 60 years, and 70 years; (2) carriers of *BRCA1/BRCA2* PV undertaking RRM at age 48 years and RRSO at age 50 years; (3) no reduction in BC risk from RRSO; (4) no HRT use or adherence; (5) half RRM uptake rate; (6) half RRSO uptake rate; and (7) lower sensitivity of *BRCA* genetic testing (97%).^49^ In the 1-way sensitivity analysis, each parameter was varied to evaluate their individual impact on results. Probabilities and utility scores were varied according to 95% CIs or ranges where available or by ±10%. Costs were varied by ±30%. Probabilistic sensitivity analysis (PSA) was undertaken, and parameters varied simultaneously across their distributions. Costs were specified as having a γ distribution; quality of life, a log-normal distribution; and probability, a β distribution, as recommended.^63^ A cost-effectiveness acceptability curve helped plot the results of 5000 simulations, showing the probability of population-based *BRCA* testing being cost-effective at different WTP thresholds in Canada. The maximum costs of genetic testing and the maximum *BRCA1/BRCA2* PV prevalence for population-based testing to remain cost-effective were explored by threshold analyses. All analyses were conducted from payer and societal perspectives.

## Results

The model simulated 1 000 000 Canadian women aged 30 years at model entry. In the base-case analysis (Table 2), the ICERs of population-based *BRCA1/BRCA2* testing compared with FH-based testing were CAD $32 276 (US $23 402.84) per QALY from the payer perspective and CAD $16 416 (US $11 903.00) per QALY from the societal perspective, well below the conventional CAD $50 000 to CAD $100 000 per QALY WTP threshold in Canada. Population-based *BRCA* testing could prevent 2555 BCs and 485 OCs per 1 000 000 Canadian population, corresponding to averting 196 BC deaths per 1 000 000 population and 163 OC deaths per 1 000 000 population during a lifetime horizon (Table 3).

**Table 2.** Lifetime Discounted Costs, Outcomes, and ICERs

Testing scenario	Health outcomes		Costs, CAD$ ($US)		ICER			
					Cost, CAD$/LY ($US)		Cost, CAD$/QALY ($US)	
	LY	QALY	Payer	Societal	Payer	Societal	Payer	Societal
**Baseline**									
FH-based^a^	34.29	34.23	3843 (2805.39)	6572 (4797.56)	NA	NA	NA	NA
Population	34.30	34.24	4186 (3055.78)	6747 (4925.31)	40 157 (29 314.61)	20 424 (14 909.52)	32 276 (23 561.48)	16 416 (11 983.68)
**Age 40 y at genetic testing; age 41 y at RRM and RRSO**									
FH-based^a^	29.18	29.12	4168 (3042.64)	6855 (5004.15)	NA	NA	NA	NA
Population	29.19	29.12	4500 (3285)	7086 (5172.78)	62 742 (45 801.66)	43 615 (31 838.95)	50 598 (36 936.54)	35 173 (25 676.29)
**Age 50 y at genetic testing; age 51 y at RRM and RRSO**									
FH-based^a^	23.41	23.34	4051 (2957.23)	6047 (4414.31)	NA	NA	NA	NA
Population	23.41	23.35	4360 (3182.8)	6259 (4569.07)	62 261 (45 450.53)	42 620 (31 112.60)	48 428 (35 352.44)	33 151 (24 200.23)
**Age 60 y at genetic testing; age 61 y at RRM and RRSO**									
FH-based^a^	16.97	16.92	2964 (2163.72)	3571 (2606.83)	NA	NA	NA	NA
Population	16.98	16.93	3263 (2381.99)	3823 (2790.79)	75 020 (54 764.60)	63 118 (46 076.14)	53 976 (39 402.48)	45 413 (33 151.49)
**Age 70 y at genetic testing; age 71 at RRM and RRSO**									
FH-based testing^a^	9.88	9.85	1878 (1370.94)	1878 (1370.94)	NA	NA	NA	NA
Population testing	9.88	9.85	2220 (1620.6)	2220 (1620.6)	635 102 (463 624.46)	635 102 (463 624.46)	269 312 (196 597.76)	269 312 (196 597.76)
**Age 49 y at RRM; age 50 y at RRSO**									
FH-based^a^	34.29	34.23	3842 (2804.66)	6577 (4801.21)	NA	NA	NA	NA
Population	34.30	34.24	4178 (3049.94)	6777 (4947.21)	46 974 (34 291.02)	27 981 (20 426.13)	37 730 (27 542.90)	22 475 (16 406.75)
**No reduction in BC risk from RRSO**									
FH-based^a^	34.29	34.23	3845 (2806.85)	6577 (4801.21)	NA	NA	NA	NA
Population	34.30	34.24	4204 (3068.92)	6790 (4956.70)	46 665 (34 065.45)	27 682 (20 207.86)	38 243 (27 917.39)	22 686 (16 560.78)
**No adherence with HRT**									
FH-based^a^	34.29	34.23	3840 (2803.20)	6570 (4796.10)	NA	NA	NA	NA
Population	34.30	34.24	4169 (3043.37)	6730 (4912.9)	41 691 (30 434.43)	20 296 (14 816.08)	32 956 (24 057.88)	16 044 (11 712.12)
**Half RRM uptake (18%)**									
FH-based^a^	34.29	34.23	3842 (2804.66)	6574 (4799.02)	NA	NA	NA	NA
Population	34.30	34.24	4184 (3054.32)	6759 (4934.07)	42 075 (30 714.75)	22 761 (16 615.53)	33 821 (24 689.33)	18 296 (13 356.08)
**Half RRSO uptake (31.4%)**									
FH-based^a^	34.29	34.23	3843 (2805.39)	6576 (4800.48)	NA	NA	NA	NA
Population	34.30	34.24	4189 (3057.97)	6772 (4943.56)	45 908 (33 512.84)	26 021 (18 995.33)	36 964 (26 983.72)	20 952 (15 294.96)
**97% Sensitivity of genetic testing**									
FH-based^a^	34.29	34.23	3843 (2805.39)	6573 (4798.29)	NA	NA	NA	NA
Population	34.30	34.24	4187 (3056.51)	6754 (4930.42)	41 543 (30 326.39)	21 810 (15 921.30)	33 390 (24 374.70)	17 530 (12 796.90)

Abbreviations: BC, breast cancer; FH, family history; HRT, hormone replacement therapy; ICER, incremental cost-effectiveness ratio; LY, life years; NA, not applicable; QALY, quality-adjusted life-year; RRM, risk-reducing mastectomy; RRSO, risk-reducing salpingo-oophorectomy.

^a^ Reference strategy.

**Table 3.** Lifetime Outcomes of Offering Genetic Testing for the Canadian Population per 1 000 000 Population^a^

Outcome	Testing strategy, events, No.		Difference, No.
	Population	FH	
BC diagnoses	95 867	98 422	2555
OC diagnoses	6341	6826	485
BC deaths	7544	7740	196
OC deaths	119	282	163
Excess CHD deaths	21	2	−19

Abbreviations: BC, breast cancer; CHD, coronary heart disease; FH, family history; OC, ovarian cancer.

^a^ Female population data are obtained from the World Bank.^44^ We used the modeling to estimate the number of BC cases, OC cases, BC deaths, OC deaths, and excess CHD deaths per million women aged 30 years, and calculated the number of cases prevented and deaths prevented.

Table 2 summarizes the scenario analyses results. Although the ICERs increased, offering genetic testing at older ages remained cost-effective, with ICERs of CAD $50 598 (US $36 687.85) per QALY (payer) and CAD $35 173 (US $25 503.41) per QALY (societal) at age 40 years; CAD $48 428 (US $35 114.42) per QALY (payer) and CAD $33 151 (US $24 037.29) per QALY (societal) at age 50 years; and CAD $53 976 (US $39 137.19) per QALY (payer) and CAD $45 413 (US $32 928.29) per QALY (societal) at age 60 years. However, it was not cost-effective in women aged 70 years (ICER of CAD $269 312 [US $195 274.09] per QALY for payer and societal perspectives). With older ages of RRM (48 years) and RRSO (50 years), population-based *BRCA1/BRCA2* testing remained cost-effective, with ICERs of CAD $37 730 (US $27 357.46) per QALY (payer) and CAD $22 475 (US $16 296.29) per QALY (societal). Even with no reduction in BC risk from RRSO, population-based *BRCA1/BRCA2* testing was cost-effective, with ICERs of CAD $38 243 (US $27 729.43) per QALY (payer) and CAD $22 686 (US $16 449.28) per QALY (societal). With no HRT use or adherence, the ICERs were CAD $32 956 (US $23 895.90) per QALY (payer) and CAD $16 044 (US $11 633.26) per QALY (societal). The ICERs increased to CAD $33 821 (US $24 523.10) per QALY (payer) or CAD $18 296 (US $13 266.16) per QALY (societal) with half RRM uptake rate, or CAD $36 964 (US $26 802.04) per QALY (payer) or CAD $20 952 (US $15 191.98) per QALY (societal) with half RRSO uptake rate, but still remained below the WTP threshold. Assuming lower sensitivity (97%) of genetic testing increased the ICERs to CAD $33 390 (US $24 210.59) per QALY (payer) and CAD $17 530 (US $12 710.74) per QALY (societal), and population-based genetic testing was still cost-effective.

The 1-way sensitivity analyses showed that model parameters, including costs, utilities, or probabilities, had little influence on base case results (eFigure 1 in Supplement 1). This included the cost of genetic testing and *BRCA1/BRCA2* prevalence, which were the variables with the maximum impact on ICERs. At the $50 000 per QALY WTP threshold, the maximum costs of genetic testing for population-based testing to remain cost-effective were CAD $410 (US $297.28) from the payer perspective and CAD $581 (US $421.27) from the societal perspective. The maximum combined *BRCA1/BRCA2* prevalence to remain cost-effective were 0.0048% from the payer perspective and 0.0039% from the societal perspective. When the WTP threshold increases to $100 000 per QALY, the maximum costs of genetic-testing for population testing to remain cost-effective were CAD $948 (US $687.38) from the payer perspective and CAD $1118 (US $810.65) from the societal perspective. The maximum combined *BRCA1/BRCA2* prevalence to remain cost-effective were 0.0029% from the payer perspective and 0.0027% from the societal perspective.

**Figure 2.**
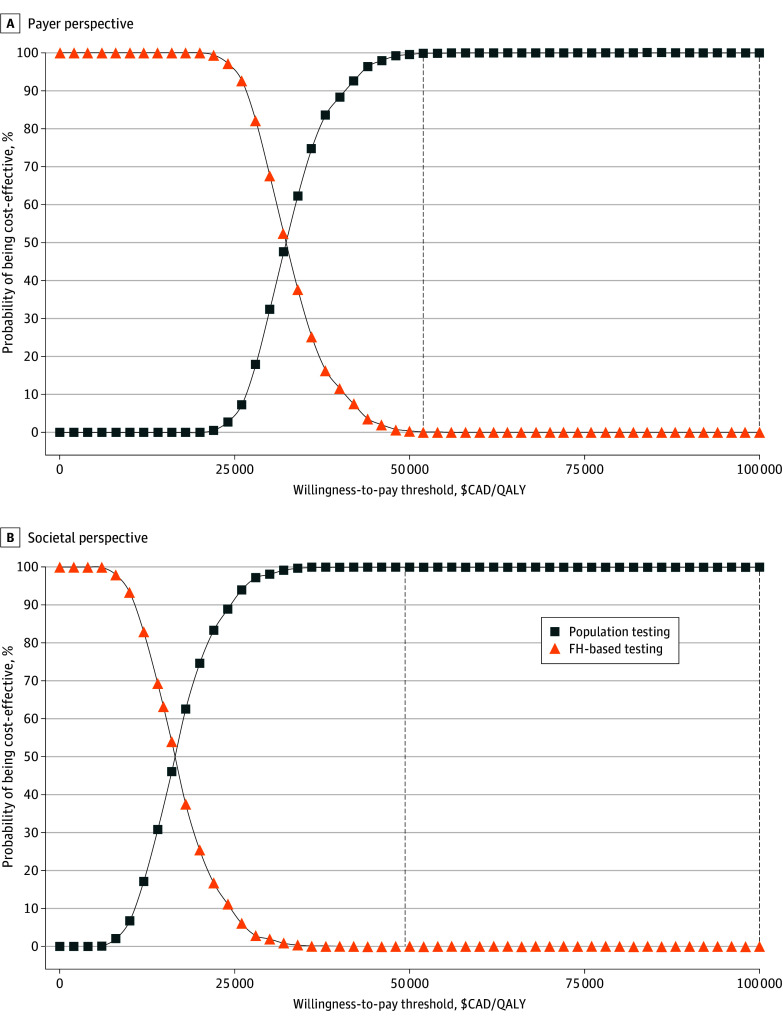
Cost-Effectiveness Acceptability Curves Using Probabilistic Sensitivity Analyses At the willingness-to-pay thresholds of CAD $50 000 (US $36 254.25) per QALY and CAD $100 000 (US $72 508.50) per QALY, 99.6% and 100% of simulations for the payer perspective were cost-effective from the payer perspective, and 100% of simulations for the societal perspective were cost-effective.

The PSA results showed that population-based *BRCA1/BRCA2* testing was highly cost-effective compared with FH-based testing (Figure 2). Overall, 99.6% of payer perspective simulations and 100% of societal perspective simulations were cost-effective at the WTP threshold of $50 000 per QALY for Canada.

## Discussion

To our knowledge, this economic evaluation is the first analysis of the cost-effectiveness of population-based *BRCA1/BRCA2* testing in Canada. Population-based *BRCA* testing was cost-effective compared with FH-based testing from payer and societal perspectives. CADTH recommends that economic evaluations use the publicly funded health care payer perspective; this may deviate depending on the decision problem.^52^ The societal perspective analysis is associated with lower ICERs per QALY (vs payer), as it incorporates additional costs linked to productivity loss.

A population-based *BRCA* testing approach can potentially prevent 2555 BCs and 485 OCs in Canada, averting 196 BC deaths and 163 OC deaths per 1 000 000 population compared with FH-based testing. Given the underutilization of *BRCA* testing with limited access and uptake associated with preventive care and treatment pathways,^11,12,27^ the benefit of a population-based genetic testing strategy could be even higher. Our findings show that a population-based approach was associated with a far greater reduction in BC/OC disease burden in the population than current treatment strategies. Cost-effectiveness analyses facilitate policy decision-making on health care resource allocation due to financial pressures within health systems. We address a key priority highlighted by a Canadian summit with respect to developing a population-based *BRCA* testing strategy for Canada.^25^ Our findings support change toward a population-based testing paradigm to maximize BC and OC prevention in Canada and highlights the need for further implementation research in this area.

Overall, our results are robust in sensitivity and scenario analyses. The cost of genetic testing has the largest impact on the cost-effectiveness of population-based testing in Canada. The cost of genetic-testing has decreased considerably over the last 10 years and remains on a downward trajectory. Our analysis of maximum costs of *BRCA* testing for a population-based strategy to remain cost-effective found that the threshold costs (payer: $410-$948; societal: $581-$1118) were largely greater than what is charged by many Canadian genetic testing laboratories today. Additionally, future costs are likely to decrease further, particularly with economies of scale.

Several health economic modeling studies have examined the cost-effectiveness of a population-based *BRCA* testing strategy across other countries both in Jewish and non-Jewish populations.^64,65,66,67,68,69,70^ In Australia, population-based *BRCA* testing for adults aged 18 to 40 years was cost-effective in combination with testing for other cancer susceptibility genes compared with FH-based testing.^69,71^ Population-based *BRCA* testing with other BC and OC genes (*PALB2*, *RAD51C*, *RAD51D*, and *BRIP1*) was cost-effective for UK and US women older than 30 years.^72^ This was similarly shown to be cost-effective for testing *BRCA1, BRCA2,* and *PALB2* in women aged 30 to 35 years in the US.^70^ Another US study reported larger ICERs for population-based multigene testing, although it was still cost-effective for women aged 30 years.^73^ Population-based genetic testing is overall cost-effective for younger women aged 30 years across multiple studies, while results may vary across other age groups and are context specific due to differences in costs of screening and surgical interventions as well as in uptake of surgical prevention strategies. These analyses, coupled with results from implementation studies among Jewish populations,^21,74,75,76,77^ have facilitated clinical implementation of population-based *BRCA* testing in Jewish populations in the UK and Israel and led to ongoing general population-based panel testing implementation studies in Australian and UK populations.^22,24^

Our analysis will enable further population-based genetic testing research in Canada. Research studies will need to develop a context-specific scalable model for the Canadian population and evaluate logistics and impact, including acceptability, satisfaction, long-term health behavior, ethical and legal considerations, and psychosocial implications with uptake rates of screening and prevention strategies. It will be equally important to address issues of equity, access, and awareness. Significant health inequalities have been observed among immigrants; sexual, racial, and ethnic minority groups, Indigenous peoples, and individuals with lower socioeconomic status or functional limitations.^78^ Another unaddressed issue is establishing a strategy for the management of VUS. While VUS are being returned and evaluated in the UK study, these are not returned in the Australian study.^22,79^

Our analysis has several advantages. We follow the transparency principle to facilitate interpretation of methods and results and use current standard-of-care or best practice as the comparator for measuring costs and health effects. Per CADTH recommendations,^52^ we use QALYs to measure health outcomes, which captures both length of life and quality of life and is generalizable across disease states. Our economic evaluation uses a lifetime horizon that is long enough to capture all costs and health outcomes relevant to the decision problem. Additionally, costs and health effects are discounted to reflect their value at time of decision-making, ensuring that potential time preferences of the relevant population are accounted for. Our base case reflects direct health care costs and health outcomes, and our analysis includes a societal perspective. We explore heterogeneity through scenario analyses and uncertainty and variability through extensive 1-way and PSA analyses, as recommended. Our results remain robust at parameter extremes on 1-way analyses. That more than 99% simulations were cost-effective with PSA adds to the robustness of the results. Besides BC and OC outcomes, excess CHD deaths from premenopausal oophorectomy, costs for HRT, bone health monitoring, and treatment are incorporated in our model. Our costs also include pretest and posttest genetic counselling (PVs and VUS).

### Limitations

Our study has some limitations. Our base case analysis incorporates a reduction for BC risk with premenopausal oophorectomy, while there has been uncertainty around this.^80^ Our scenario analysis shows the cost-effectiveness of a population-based testing strategy in Canada without BC risk reduction from RRSO. The uptake rates of surgical prevention could be lower in carriers of *BRCA1/BRCA2* PVs identified from population-based testing, particularly in the absence of cancer within the family. Decision-making for undergoing preventive surgery can be complex, is affected by fertility wishes, impact of menopause, and changes with time.^81^ We did not model a nonconstant, age-based uptake in the model, and that can be a limitation. However, we explored this through our scenario analyses, which confirmed the cost-effectiveness of population-based testing from payer and societal perspectives, with half of the base case surgical prevention uptake rate, as well as for older ages of preventive surgery until age 61 years, although the ICER per QALY increased with increasing ages. More prospective data on age-based uptake rates of surgical prevention strategies following population-based testing are needed for Canada. Although we include a disutility for RRSO and RRM in the analysis, these procedures have potential complication rates of approximately 3% to 4% and 21%, respectively.^82,83^ While RRSO has been reported to alleviate cancer distress and worry and has high acceptability and satisfaction rates (>85%),^84^ poorer sexual function and increased menopause symptoms despite HRT use have been reported.^85,86^ The RRSO decision regret rate is higher in premenopausal (9%) than postmenopausal (1%) women.^85,87^ RRM has an adverse association with body image and sexual function (eg, frequency, sensation, pleasure) but not with anxiety or depression or generic quality of life, and overall satisfaction rates are good.^86,88^ These issues need to be part of the informed consent and decision-making process. Additionally, while we undertook sensitivity analysis for disutilities associated with BC and OC treatment, more up-to-date estimates are needed for different stages of disease. While productivity loss was included in our analysis, we did not include all indirect costs. This may be a limitation; however, including additional indirect costs would further improve the cost-effectiveness of population-based testing, so our analysis is conservative in that respect. Our modeling analysis does not include of *BRCA* carriers who may have already been identified through FH-based testing in the population through current clinical practices. Thus, our approach is conservative, as incorporating this will decrease the beneficial impact of FH testing, making population testing even more cost-effective, as there is no change in the identification of the *BRCA* carriers without FH. Additionally, we have previously reported that 97% of *BRCA* carriers in the general population remain unidentified despite 30 years of FH-based *BRCA* testing,^12^ minimizing the impact of this issue. Population-based testing would even identify individuals with an FH who should have been detected through clinical routes but have been missed and may opt for a population program if offered. An important issue is whether population-based *BRCA* testing could lead to false reassurance, given that most individuals will have negative test results, and have a detrimental impact on lifestyle behaviors, such as smoking, alcohol consumption, diet, physical exercise, and routine mammography screening. A randomized population-based *BRCA* testing trial in an Ashkenazi Jewish population did not show a negative association in any of the aforementioned lifestyle behaviors in participants whose test results were negative for *BRCA*.^89^ Hence, we did not include a detriment for this in our base case analysis. However, more prospective general population data are needed on this important issue. Furthermore, *BRCA* testing was limited in this model to women only. Men are also at risk for *BRCA*-associated cancers, including high-risk prostate cancer, although the lifetime risk is lower than for BC and OC in women. However, the downstream benefit of testing men would be realized through cascade testing and preventing BC and OC in family members.

## Conclusions

The findings of the economic evaluation support the potential cost-effectiveness of *BRCA1/BRCA2* genetic testing on a broader scale in the Canadian general population, which could prevent thousands more BC and OC diagnoses and deaths than FH-based testing. Such an approach could bring about a new paradigm for improving global cancer prevention. The increasing public awareness and acceptability of genetic testing and decreasing costs, coupled with computational and technological advancements, provide the ability to implement large-scale population-based genetic testing for actionable tier 1 genes, like *BRCA1* and *BRCA2*. Context-specific implementation strategies and pathways for population-based genetic testing need to be developed. Implementation studies providing data on the impact of population-based *BRCA* testing under real-world settings are ongoing, including ongoing project surveys of the Canadian population about preferences and ideal implementation models. This is essential for population genomics to achieve its potential for maximizing cancer prevention.

## Notes

Supplement 1. **eMethods 1.** Explanation of the Markov Model Structure **eTable 1.** Probability Values and Explanations **eTable 2.** Medical Costs in 2022 Values and Explanations **eMethods 2.** Estimation of Productivity Loss **eMethods 3.** Estimates for Age of Onset and Survival for Breast and Ovarian Cancers **eFigure.** Tornado Diagrams of 1-Way Sensitivity Analyses
eReferences.

Supplement 2.
Data Sharing Statement
