# Supplementary material for: Economic Evaluation of Population-Based BRCA1 and BRCA2 Testing in Canada
Source: JAMA Netw Open. 2024 Sep 12;7(9):e2432725. doi: 10.1001/jamanetworkopen.2024.32725 (PMC11393724; doi:10.1001/jamanetworkopen.2024.32725)
Supplement: Supplement 1. — eMethods 1. Explanation of the Markov Model Structure eTable 1. Probability Values and Explanations eTable 2. Medical Costs in 2022 Values and Explanations eMethods 2. Estimation of Productivity Loss eMethods 3. Estimates for Age of Onset and Survival for Breast and Ovarian Cancers eFigure. Tornado Diagrams of 1-Way Sensitivity Analyses eReferences. [file jamanetwopen-e2432725-s001.pdf]

Supplemental Online Content

Sun L, Wei X, Fierheller CT, et al. Economic evaluation of population-based *BRCA1* and *BRCA2* testing in Canada. *JAMA Netw Open*. 2024;7(9):e2432725. doi:10.1001/jamanetworkopen.2024.32725

eMethods 1. Explanation of the Markov Model Structure
eTable 1. Probability Values and Explanations
eTable 2. Medical Costs in 2022 Values and Explanations
eMethods 2. Estimation of Productivity Loss
eMethods 3. Estimates for Age of Onset and Survival for Breast and Ovarian Cancers
eFigure. Tornado Diagrams of 1-Way Sensitivity Analyses
eReferences.

This supplemental material has been provided by the authors to give readers additional information about their work.

© 2024 Sun L et al. *JAMA Network Open.*

eMethods 1. Explanation of the Markov model structure

Figure 1 includes the choice between "Population based testing" and "Clinical criteria/ family-history (FH) testing", and the schematic illustration of the health states and key transitions for the Markov model. Progression through the model is dependent on the probabilities provided in Table 1.

In the population testing arm, all women 30 years old are offered *BRCA1/BRCA2* testing and get classified as *BRCA* positive (i.e., pathogenic variant (PV) carriers) or *BRCA* negative. A small proportion (2%) may also have variants of uncertain significance (VUS).[1] All individuals receive pre-test counselling and post-test counselling is provided to *BRCA* PV carriers and those with VUS. *BRCA* PV carriers identified are offered options of risk-reducing mastectomy (RRM) and risk-reducing salpingo-oophorectomy (RRSO). Depending on the probability of *BRCA* women undertaking RRM and/or RRSO (+/- medical prevention) they are placed into different health states and then progress to either *BRCA* associated breast cancer (BC) or *BRCA* associated ovarian cancer (OC). All women undergoing RRSO have an increased risk of fatal coronary heart disease (CHD). They also have a probability of dying from the background all-cause mortality. A small proportion of VUS may get reclassified as *BRCA* PV carriers in the future and this is incorporated into the model. These individuals may then access all options of screening and prevention routinely available to *BRCA* PV carriers. *BRCA* positive women who do not progress or die would stay in the health states and undertake the next cycle. *BRCA1/BRCA2* PV negative women progress to sporadic non-*BRCA* OC or non-*BRCA* BC based on the age-dependent probabilities. They also have a probability of dying from the background all-cause mortality. Women who do not progress or die stay in the health states to undertake the next cycle.

In the FH arm, only women with FH that fulfils current clinical-criteria (based on current guidelines) undergo *BRCA1/BRCA2* genetic testing and get classified as *BRCA* positive or *BRCA* negative. A small proportion may also have VUS.[1] All individuals receive pre-test counselling and post-test counselling is provided to *BRCA* PV carriers and those with VUS. Women with a negative FH are either *BRCA* negative or have an undetected *BRCA* PV. Options of RRM and RRSO and disease progression for identified *BRC*A PV carriers and disease progression for *BRCA* negative women, are the same as those in the population testing arm and are described above. All women undergoing RRSO have an increased risk of CHD. Undetected *BRCA* women are not offered RRM or RRSO. Depending on the baseline risk (no risk-reducing options) they progress to *BRCA* associated BC or *BRCA* associated OC. They also have a probability of dying from the background all-cause mortality. A small proportion of VUS may get reclassified as *BRCA* PV carriers in the future and this is incorporated into the model. These individuals may then access all options of screening and prevention routinely available to *BRCA* PV carriers. Women who do not progress or die stay in the health state of *BRCA* undetected and undertake the next cycle.

© 2024 Sun L et al. *JAMA Network Open.*

eTable 1. Probability values and explanations

Probability	Description	Value	(95% CI) [Range]	Source
P1	*BRCA1/2* PV prevalence in general population	0.0067	(0.0059, 0.0077)	[2]
P2	Probability that *BRCA1/2* PV carriers will undergo RRM	0.359	(0.287, 0.431)	[3]
P3	Reduction in ovarian cancer risk from RRSO	0.96	[0.8, 0.96]	[4,5]
P4	Probability of having a positive FH	0.0098	(0.0047, 0.0179)	ABCFS
P5	*BRCA1/2* PV prevalence in FH positive individuals	0.1	--	[6]
P6	*BRCA1/2* PV prevalence in FH negative individuals	0.0058	(0.0051, 0.0068)	[2], ABCFS
P7	Reduction in breast cancer risk from RRM without RRSO in *BRCA1/2* PV carriers	0.91	(0.62, 0.98)	[7]
P8	Probability that *BRCA1/2* PV-carriers will undergo RRSO	0.628	(0.502, 0.754)	[8]
P9	HR in breast cancer risk from RRSO alone	0.49	(0.37,0.65)	[5]
P10	Reduction in risk of breast cancer from RRM with RRSO	0.95	(0.78, 0.99)	[7]
P11	Excess CHD risk	0.0072	(0.0068, 0.0076)	[9]
P12	Fatal CHD risk	0.0303	(0.011, 0.043)	[9]
P13	Compliance with HRT	0.8	(0.76, 0.83)	[10]
P14	HR of breast cancer risk from breast cancer chemoprevention	0.71	(0.6, 0.83)	[11]
P15	Uptake of breast cancer chemoprevention	0.086	(0.069, 0.103)	[3]

95%CI – 95% confidence interval, ABCFS - Australia Breast Cancer Family Study, CHD – coronary heart disease, FH – family history, HR – hazard ratio; HRT – hormone replacement therapy; PV – pathogenic variant; RRM – risk-reducing mastectomy, RRSO – risk-reducing salpingo-oophorectomy

P1: *BRCA1/2* PV prevalence in general population is calculated based on Jervis 2015.[2]
P2: The probability that unaffected *BRCA1/2* PV-carriers will undergo RRM is taken from an international study on uptake rates of cancer risk-reduction strategies for *BRCA1/2 PV*-carriers by Metcalfe et al 2019,[3] and the Canadian uptake rate was used in the model.
P3: The reduction in ovarian cancer risk obtained from RRSO is taken from previous studies which report a 4% residual-risk of primary peritoneal cancer following RRSO.[4]
P4: The probability of having a positive family history in general population is obtained from the Australia Breast Cancer Family Study (ABCFS).
P5: The overall *BRCA1/BRCA2* PV prevalence (10%) among FH positive breast cancer patients is based on the current testing guideline.[6]
P6: The *BRCA1/2* PV prevalence in FH negative individuals is calculated based on the *BRCA1/2* PV prevalence in general population, the *BRCA1/2* PV prevalence in FH positive individuals, and the probability of having a positive FH.
P7: The reduction in breast cancer risk from RRM in *BRCA1/2* PV-carriers not undergoing RRSO is taken from the PROSE study by Rebbeck et al 2004.[7]
P8: The probability that unaffected *BRCA1/BRCA2* PV-carriers will undergo RRSO is taken from a British Columbia study by Hanley et al 2019.[8] A composite uptake rate for *BRCA1* (64.7% RRSO rate) and *BRCA2* (62.2% RRSO rate) weighted for the relative prevalence of *BRCA1* and *BRCA2* PV was computed.
P9: The Hazard Ratio for breast cancer in pre-menopausal unaffected *BRCA1/2* PV-carriers undergoing RRSO alone is taken from a meta-analysis by Rebbeck et al 2009.[5]
P10: The reduction in breast cancer risk in *BRCA1/2* PV-carriers undergoing RRM and RRSO is taken from the PROSE study by Rebbeck et al 2004.[7]
P11: Excess risk of CHD after RRSO is estimated using data from Parker 2013.[9] The absolute excess CHD incidence is obtained by subtracting CHD incidence in women undergoing RRSO from those not.
P12: The risk of CHD mortality is obtained from the Nurses' Health Study (Parker et al 2013).[9] Death from CHD is reported in 1 in 33 pre-menopausal women undergoing RRSO and not taking HRT.[9]
P13: HRT compliance rate is obtained from a UK cohort (Read et al, 2010).[10]
P14: The Hazard Ratio for breast cancer risk from chemoprevention in high-risk women is obtained from the extended long-term follow-up of the IBIS-I breast cancer prevention trial (Cuzick et al 2015).[11]
P15: The uptake of breast cancer chemoprevention is obtained from study by Metcalfe et al 2019.[3]

© 2024 Sun L et al. *JAMA Network Open.*

eTable 2. Medical costs in 2022 values and explanations

Cost descriptions	Values (CAD)
Cost of genetic testing	220
Cost of genetic counselling	167
Cost of risk-reducing salpingo-oophorectomy	4901
Cost of ovarian cancer diagnosis and treatment	21800
Annual cost of ovarian cancer in years 1 to 2	7300
Annual cost of ovarian cancer in years 3 to 5	7010
Terminal care cost with ovarian cancer	52697
Cost of risk-reducing mastectomy	12330
Annual cost of hormone replacement therapy	680
Cost of mammography	144
Cost of MRI	130
Cost of breast cancer diagnosis and treatment in general population	33155
Annual cost of breast cancer in general population	1414
Cost of breast cancer diagnosis and treatment in *BRCA1/2* PV-carriers	33155
Annual cost of breast cancer in *BRCA1/2* PV-carriers	1284
Terminal care cost with breast cancer	43638
Cost of fatal coronary heart disease	4839
Annual cost of excess coronary heart disease	175
Annual cost of chemoprevention	293

CAD- Canadian Dollars, CHD – cornorary heart disease, HRT – hormone replacement therapy, MRI - magnetic resonance imaging, RRM – risk-reducing mastectomy, RRSO – risk-reducing salpingo-oophorectomy, PPP- purchasing power parity.

Explanations:
All costs are reported at 2022 Canadian dollars.

Cost of genetic testing/counselling
We use a standard international cost for genetic testing used in Canada (CAD$ 220 in 2022), and assume a 100% uptake of genetic testing.

The cost of *BRCA1/BRCA2* testing is based on testing costs for these genes taken from a Canadian programme.[12] The Canadian national unit cost assumed for genetic counselling is $500 per hour which includes overhead, facility, and administrative costs. All participants have pre-test counselling and post-test counselling is received by those testing positive (pathogenic/likely-pathogenic carriers) as well as those with VUS.

Costs of RRSO and RRM
The RRSO costs are obtained from the Medical Services Commission Payment Schedule in Canada[13] and the Canadian Institute for Health Information Patient Cost Estimator.[14] Costs of HRT are taken from Medical Services Commission Payment Schedule,[13] which are dependent on health insurance status and province. HRT is given from the average age of RRSO to the average age of menopause (51 years). These costs are calculated for the 80% assumed to be compliant with HRT. RRSO costs also include the cost of three follow up DEXA scans for monitoring bone health and calcium and vitamin-D3 for additional osteo-protection.

The RRM costs are obtained from the Medical Services Commission Payment Schedule in Canada[13] and the Canadian Institute for Health Information patient cost estimator.[14]

Costs of ovarian cancer
We assume that the costs of ovarian cancer diagnosis include a pelvic examination, ultrasound scan, CA125 test, CT scan, percutaneous biopsy, and peritoneal cytology. The costs of ovarian cancer treatment include the cost for a lower and upper genital tract very complex major procedure and administration of chemotherapy based on

© 2024 Sun L et al. *JAMA Network Open.*

6 cycles of carboplatin and paclitaxel treatment. It is assumed that in the first and second years treated survivors would have a further three consultant visits, a CT scan and four CA125 tests each year. In the third to fifth years post-surgery, it is assumed that survivors would have two consultant visits and two CA125 tests.

Costs for ovarian cancer diagnosis and treatment in Canada are obtained from the Medical Services Commission Payment Schedule in Canada[13] and the Canadian Institute for Health Information Patient Cost Estimator.[14] We include the treatment costs of recurrence of ovarian cancer, taken from a Canadian inter-provincial comparison study for the cost of cancer care in British Columbia and Ontario.[15] The costs of ovarian cancer terminal care are derived from the terminal phase (12 months) costs in British Columbia and Ontario.[15]

Costs of breast cancer

In the general population, 10% breast cancer is non-invasive ductal carcinoma in situ (DCIS) and 90% is invasive. Among invasive breast cancers, 46.6%, 35.1%, 12.4%, and 4.9% are stage 1, stage 2, stage 3, and stage 4 breast cancers, respectively.[16] We assumed the same stage distribution for *BRCA1/BRCA2* PV carriers as that for general population due to lack of data. In the general population, 64.8% of invasive breast cancers are ER-positive,[17] among which 49% are premenopausal. 13.1% of early/locally advanced breast cancers and 22.2% of advanced breast cancers are HER2-positive. 27% *BRCA1* and 67% *BRCA2* breast cancers are ER-positive; 5% *BRCA1* and 14% *BRCA2* breast cancers are HER2-positive.[18-23] All costs are adjusted for *BRCA1/BRCA2* breast cancers for differences in stage at presentation, the proportion of being non-invasive, and the proportion of being ER-positive or HER2-positive.

Breast cancer treatment costs in Canada are estimated based on clinical guidelines and unit costs detailed as below.

Diagnosis costs: The breast cancer diagnosis costs including mammography, MRI, ultrasound imaging with core biopsy and/or fine needle aspiration cytology costs, are taken from the Medical Services Commission Payment Schedule in Canada.[13] For all patients presented with suspected advanced breast cancer, MRI should be offered to assess for bone metastases.[24]

Sentinel lymph node biopsy (SLNB) costs: SLNB is used for staging axilla for early invasive breast cancer and no evidence of lymph node involvement on ultrasound or a negative ultrasound-guided needle biopsy (73% of early and locally advanced invasive cancers). The SLNB costs are obtained from the Medical Services Commission Payment Schedule in Canada,[13] including sentinel lymph node scan and unilateral intermediate breast procedures.

Pre-treatment axilla ultrasound costs: Pre-treatment ultrasound evaluation of the axilla should be performed for all patients being investigated for early invasive breast cancer and, if morphologically abnormal lymph nodes are identified, ultrasound-guided needle sampling should be offered. The commissioning cost of pre-treatment ultrasound evaluation of the breast and axilla is the same as that of the breast only. The costing model considers the cost of ultrasound-guided needle sampling only, obtained from the Medical Services Commission Payment Schedule in Canada.[13]

Axillary lymph node dissection (ALND) costs: ALND is undertaken for lymph node positive cancers (~31% early and locally advanced invasive cancers - NICE guideline and BCCOM project;[25-27] 30% node positive for *BRCA1/2* breast cancer- familial breast cancer screening studies, breast cancer case series and Early Breast Cancer Trialists' Collaborative Group data.[18-20,28,29]) Cost of ALND is assumed to be 25% of the cost of breast surgery as per UK NICE guideline development group recommendation.[26]

Breast surgery costs include costs of breast conserving surgery (assumed for all non-invasive cancers, and 75% of early/locally advanced invasive cancers) and costs of mastectomy with reconstruction (for 25% early/locally advanced and all advanced cancers). Costs are obtained from the Medical Services Commission Payment Schedule in Canada[13] and the Canadian Institute for Health Information Patient Cost Estimator.[14]

Chemotherapy and radiotherapy costs: Invasive breast cancers who are not at low risk receive adjuvant treatment.[30] Costs include radiotherapy costs for 60% of early invasive/locally advanced, radiotherapy and chemotherapy costs for 40% early invasive/locally advanced, and chemotherapy for all advanced cancers. Radiotherapy costs include planning and 40Gy in 15 fractions over 3 weeks[30] or palliative treatment, taken from national UK NHS reference costs[31]. Chemotherapy costs based on polychemotherapy,[32] include administration costs, costs of 1st and 2nd line therapy and toxicity from the Medical Services Commission Payment Schedule in Canada[13] and the Canadian Institute for Health Information Patient Cost Estimator.[14]

© 2024 Sun L et al. *JAMA Network Open.*

Endocrine therapy costs: ER-positive invasive breast cancers receive Tamoxifen 20mg/day (premenopausal) or Anastrazole 1mg/day (postmenopausal).[30] 64.8% of invasive breast cancers are ER-positive,[17] among which 49% are premenopausal. We assume the length of endocrine therapy is 5 years. The drug and ER testing costs are obtained from a previous study[33] and included for all invasive breast cancers.

Target therapy costs: HER2-positive breast cancer patients can be given target therapy for 1 year or until disease recurrence.[30] Breast cancer patients with positive HER2 are eligible for treatment with trastuzumab. 10% of the eligible patients are intolerant of trastuzumab. Among women suitable for this treatment, 80% receive trastuzumab.[26] HER2 testing costs are obtained from the Medical Services Commission Payment Schedule in Canada[13] and included for all invasive breast cancers. The trastuzumab cost per patient including administration of treatment and cardiac monitoring is $3,466.55 per month, obtained from the pan-Canadian Oncology Drug Review.[34]

Follow up costs: Breast cancer patients are offered mammographic surveillance and clinical follow-up, whose costs are taken from the Medical Services Commission Payment Schedule in Canada.[13] We assume patients are followed up every four months in the first two years, every six months from the third to the fifth year, and every year from the sixth to the tenth year.

Bisphosphonate costs: Bisphosphonates is considered to be offered to patients newly diagnosed with bone metastases, to prevent skeletal-related events and reduce pain.[24] 74% patients with advanced breast cancer will develop bone metastases and 65% patients with bone metastases are offered bisphosphonates.[26,35] Bisphosphonates that are currently offered include oral sodium clodronate, ibandronic acid, zoledronic acid, and pamidronate. The proportions of patients receiving the four drugs are 20%, 30%, 25%, and 25% respectively. The annual costs including administration for the four drugs are £1971, £2541.96, £3208, and £3208 respectively, obtained from NICE costing report [26]. We assume the average length of bisphosphonates treatment is 2.7 years, which is the life expectancy of advanced breast cancers based on one-year survival rate (63.2%).[36]

Recurrence costs: For non-invasive breast cancers, the non-invasive and invasive relapse rates are both 12.5%. 35% of early and locally advanced invasive breast cancers progress to advanced disease.[26] The recurrence rates for early and locally advanced breast cancer are 15.9% for node-positive[37] and 11% for node-negative disease[38]. Weighted for 31% node positive and 69% node negative, the composite recurrence rate for early and locally advanced breast cancer is 12.5%. The recurrence rate for the advanced disease is 66% (34% relapse-free five-year survival).[39]

Terminal care costs: The costs of breast cancer terminal care are derived from the terminal phase (12 months) costs in British Columbia and Ontario.[15]

Cost of breast cancer screening

For non-carriers or undetected carriers, breast screening in Canada assumes mammography every two years from age 50 to 74 years (12 mammograms on average).[40]

For *BRCA1/BRCA2* PV carriers detected, we assume annual mammogram from 40-69 years and annual MRI from 30-49 years as per NICE guidelines for familial breast cancer (30 mammograms and 20 MRIs on average).[41]

Cost of chemoprevention

BRCA1/BRCA2 PV carriers are offered Tamoxifen (premenopausal) or Raloxifene (postmenopausal) for 5 years[41,42] to reduce breast cancer risk. The drug costs are obtained from the pan-Canadian Oncology Drug Review.[34] An 8.6% uptake is assumed for chemoprevention.[3]

Cost of CHD

Cost of excess CHD: The annual cost of excess CHD event is taken from study in Alberta by Tran et al,[43] with the onset of CHD estimated at 55 years of age.[9,44] This yearly cost is multiplied by the number of years between onset of CHD and average life expectancy to provide the cost attributed to excess CHD.

Cost of fatal CHD: This cost is obtained from Nova Scotia Health system.[45]

© 2024 Sun L et al. *JAMA Network Open.*

eMethods 2. Estimation of productivity loss

The retirement ages for women is 65 in Canada and the female labour force participation rate is 66.9%. The hourly wage rates are presented in the table below.

Age	Hourly wage rates
25-54	31.95
55-64	29.74
Source	Statistics Canada

We categorised the productivity costs as three subcomponents: 1) temporary disability due to short-term work absences following diagnosis, 2) permanent disability due to reduced working hours following a return to work or workforce departure; and 3) premature mortality due to death before retirement,[46] detailed below.

Descriptive statistics for productivity loss in breast and ovarian cancer patients

Variables	Breast cancer	Ovarian cancer
**(1) Temporary disability**		
Percentage of temporary disability cases	94.0%	98%[1]
Average time taken off work following diagnosis (weeks)	44.9	47.22[2]
**(2) Permanent disability**		
Percentage of permanent disability: reduced hours	26%	40%[3]
Reduced hours per week after returning to work (hours)	5.5	5.5
**(3) Premature mortality (before retirement)**		
Percentage of permanent disability: workforce departure	12.9%	30%[3]

Source: Hanly P et al, 2012 [46]

[1] We assume 98% ovarian cancer patients have cancer-related short-term work absences after diagnosis.
[2] We assume ovarian cancer patients experience four weeks for surgery, 24 weeks for chemotherapy, and 24 weeks for recurrence treatment with the recurrence rate of 80%.[47]
[3] We assume the percentages of permanent disability for ovarian cancer are 40% for reduced working hours and 30% for workforce departure.

We estimated temporary disability as time absent from work multiplied by age-specific gross earnings.

We calculated productivity costs due to permanent disability by applying age-specific gross earnings to the reduction in working hours, or the number of working hours if permanent workforce departure, until retirement age. Regarding productivity loss from premature mortality, we assumed that without cancer, the productive capacity of an individual would continue from the age of diagnosis until age of retirement. We multiplied the projected years of life lost by the age-specific gross earnings for the remainder of the working life to generate monetary estimates.

© 2024 Sun L et al. *JAMA Network Open.*

eMethods 3. Estimates for age of onset and survival for breast and ovarian cancers

Our analysis incorporates lifetime risks and long-term consequences providing a lifetime time-horizon. Female lifetables obtained from Statistics Canada[48] were used for life expectancy by 80 years for women not developing OC/BC.

We assumed that the median age for undergoing RRM and RRSO in unaffected *BRCA1/2* PV carriers was 37 and 40 years respectively.[49] We explored 48 years for RRM and 50 years for RRSO in our scenario analysis. The uptake rates of RRSO and RRM were obtained from established literature.[3,8] OC/BC outcomes were modelled using 5-year survival data from Canadian Cancer statistics 2022[50] and published literature.[51,52] No significant survival differences between hereditary (*BRCA1* and *BRCA2* PV) and sporadic BC/OC have been found.[53-55] For BC, the 5-year survival rates is 89% and for OC the 5-year survival rate is 44%. After five-years, we assumed the probability of death for all OC/BC patients was same as the general population. The excess risk of CHD following premenopausal oophorectomy is incorporated in the analysis.[9,56]

© 2024 Sun L et al. *JAMA Network Open.*

eFigure 1. Tornado diagrams of 1-way sensitivity analyses

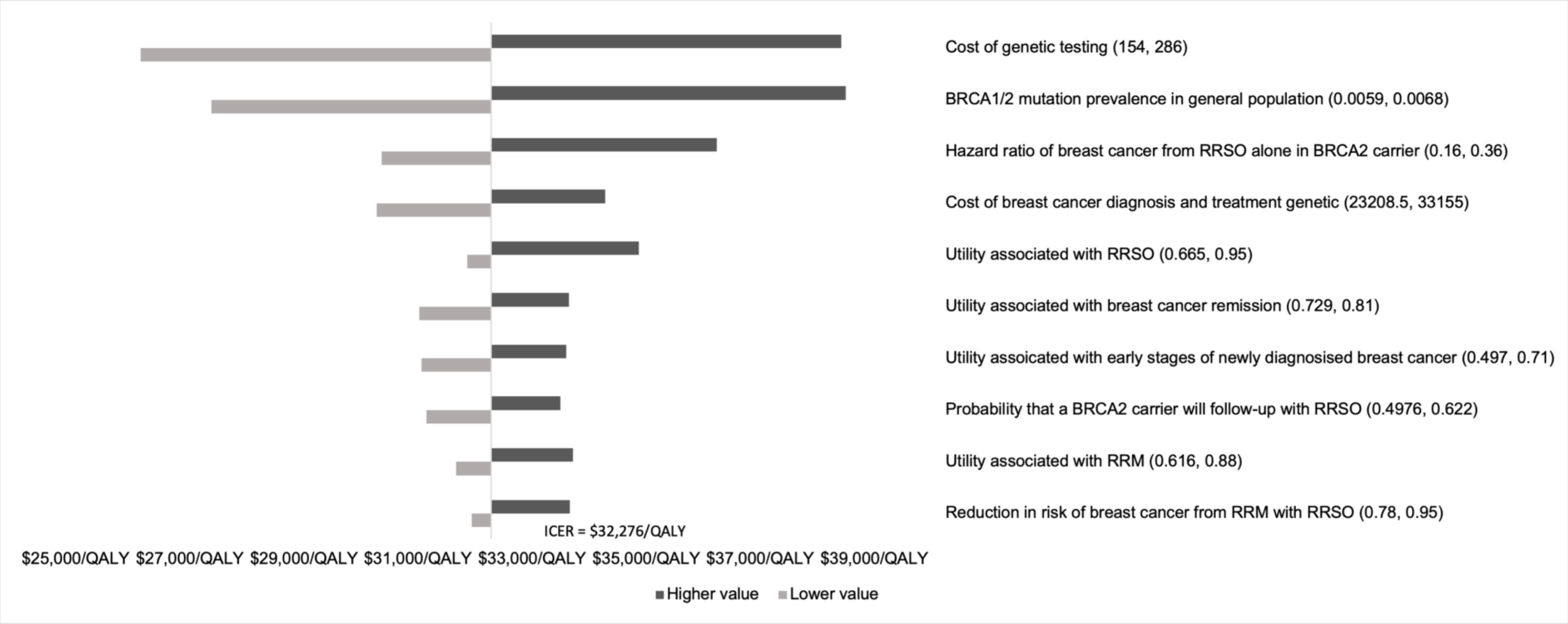

1a. Tornado diagram – payer perspective

© 2024 Sun L et al. *JAMA Network Open.*

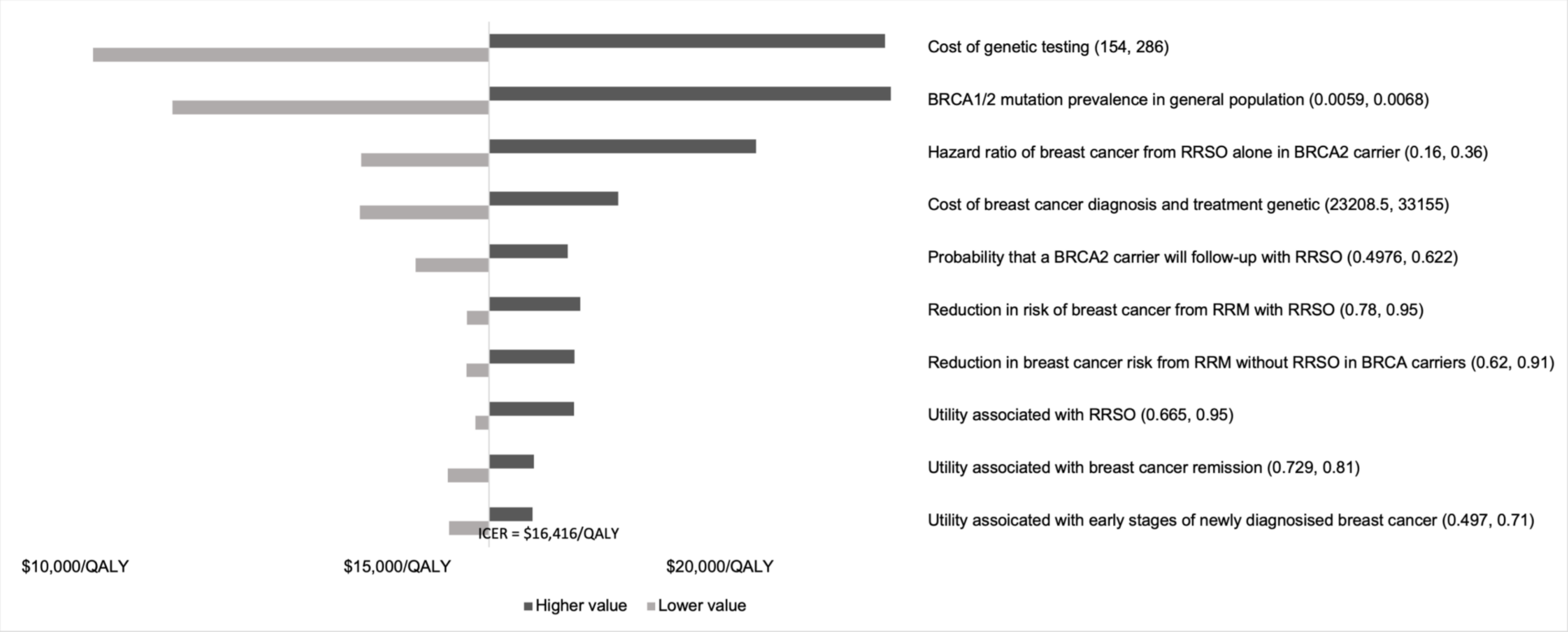

1b. Tornado diagram – societal perspective

© 2024 Sun L et al. *JAMA Network Open.*

eReferences

1. Eggington JM, Bowles KR, Moyes K, et al. A comprehensive laboratory-based program for classification of variants of uncertain significance in hereditary cancer genes. *Clin Genet*. Sep 2014;86(3):229-37. doi:10.1111/cge.12315
2. Jervis S, Song H, Lee A, et al. A risk prediction algorithm for ovarian cancer incorporating BRCA1, BRCA2, common alleles and other familial effects. *J Med Genet*. Jul 2015;52(7):465-75. doi:10.1136/jmedgenet-2015-103077
3. Metcalfe K, Eisen A, Senter L, et al. International trends in the uptake of cancer risk reduction strategies in women with a BRCA1 or BRCA2 mutation. *Br J Cancer*. Jul 2019;121(1):15-21. doi:10.1038/s41416-019-0446-1
4. Finch A, Beiner M, Lubinski J, et al. Salpingo-oophorectomy and the risk of ovarian, fallopian tube, and peritoneal cancers in women with a BRCA1 or BRCA2 Mutation. *JAMA*. Jul 12 2006;296(2):185-92. doi:10.1001/jama.296.2.185
5. Rebbeck TR, Kauff ND, Domchek SM. Meta-analysis of risk reduction estimates associated with risk-reducing salpingo-oophorectomy in BRCA1 or BRCA2 mutation carriers. *J Natl Cancer Inst*. Jan 21 2009;101(2):80-7. doi:10.1093/jnci/djn442
6. *Familial breast cancer: classification, care and managing breast cancer and related risks in people with a family history of breast cancer*. 2019. *National Institute for Health and Care Excellence: Guidelines*.
7. Rebbeck TR, Friebel T, Lynch HT, et al. Bilateral prophylactic mastectomy reduces breast cancer risk in BRCA1 and BRCA2 mutation carriers: the PROSE Study Group. *J Clin Oncol*. Mar 15 2004;22(6):1055-62. doi:10.1200/JCO.2004.04.188
8. Hanley GE, McAlpine JN, Cheifetz R, Schrader KA, McCullum M, Huntsman D. Selected Medical Interventions in Women With a Deleterious Brca Mutation: A Population-Based Study in British Columbia. *Current Oncology*. 2019;26(1):17-23.
9. Parker WH, Feskanich D, Broder MS, et al. Long-term mortality associated with oophorectomy compared with ovarian conservation in the nurses' health study. Comparative Study
Research Support, N.I.H., Extramural. *Obstet Gynecol*. Apr 2013;121(4):709-16. doi:10.1097/AOG.0b013e3182864350
10. Read MD, Edey KA, Hapeshi J, Foy C. Compliance with estrogen hormone replacement therapy after oophorectomy: a prospective study. Research Support, Non-U.S. Gov't. *Menopause international*. Jun 2010;16(2):60-4. doi:10.1258/mi.2010.010023
11. Cuzick J, Sestak I, Cawthorn S, et al. Tamoxifen for prevention of breast cancer: extended long-term follow-up of the IBIS-I breast cancer prevention trial. *Lancet Oncol*. Jan 2015;16(1):67-75. doi:10.1016/S1470-2045(14)71171-4
12. Narod SA, Gojska N, Sun P, et al. The Screen Project: Guided Direct-To-Consumer Genetic Testing for Breast Cancer Susceptibility in Canada. *Cancers (Basel)*. Apr 15 2021;13(8)doi:10.3390/cancers13081894
13. Ministry of Health. *Medical Services Commission Payment Schedule* 2021. Accessed 13 April 2023. https://www2.gov.bc.ca/assets/gov/health/practitioner-pro/medical-services-plan/msc-payment-schedule-may-2021.pdf
14. Canadian Institute for Health Information. *Patient Cost Estimator*. CIHI. https://www.cihi.ca/en/patient-cost-estimator
15. De Oliveira C, Pataky R, Bremner KE, et al. Estimating the Cost of Cancer Care in British Columbia and Ontario: A Canadian Inter-Provincial Comparison. *Healthc Policy*. Feb

© 2024 Sun L et al. *JAMA Network Open.*

2017;12(3):95-108. Estimation du coût des soins contre le cancer en Colombie-Britannique et en Ontario: une comparaison interprovinciale au Canada.
16. Staitstics Canada. *Cancer in Canada: Stage at diagnosis*. 2018. https://www150.statcan.gc.ca/n1/pub/82-003-x/2018012/article/00003/c-g/c-g03-eng.htm
17. Seung SJ, Traore AN, Pourmirza B, Fathers KE, Coombes M, Jerzak KJ. A population-based analysis of breast cancer incidence and survival by subtype in Ontario women. *Curr Oncol*. Apr 2020;27(2):e191-e198. doi:10.3747/co.27.5769
18. Cortesi L, Turchetti D, Marchi I, et al. Breast cancer screening in women at increased risk according to different family histories: an update of the Modena Study Group experience. *BMC cancer*. 2006;6:210.
19. Leach MO, Boggis CR, Dixon AK, et al. Screening with magnetic resonance imaging and mammography of a UK population at high familial risk of breast cancer: a prospective multicentre cohort study (MARIBS). Comparative Study
Multicenter Study
Research Support, Non-U.S. Gov't. *Lancet*. May 21-27 2005;365(9473):1769-78. doi:10.1016/S0140-6736(05)66481-1
20. Robson ME, Chappuis PO, Satagopan J, et al. A combined analysis of outcome following breast cancer: differences in survival based on BRCA1/BRCA2 mutation status and administration of adjuvant treatment. *Breast cancer research : BCR*. 2004;6(1):R8-R17. doi:10.1186/bcr658
21. Comen E, Davids M, Kirchhoff T, Hudis C, Offit K, Robson M. Relative contributions of BRCA1 and BRCA2 mutations to "triple-negative" breast cancer in Ashkenazi Women. *Breast Cancer Res Treat*. Aug 2011;129(1):185-90. doi:10.1007/s10549-011-1433-2
22. Tung N, Garber JE, Lincoln A, Domchek SM. Frequency of triple-negative breast cancer in BRCA1 mutation carriers: comparison between common Ashkenazi Jewish and other mutations. Comment
Letter. *J Clin Oncol*. Dec 10 2012;30(35):4447-8. doi:10.1200/JCO.2012.44.5635
23. Chappuis PO, Nethercot V, Foulkes WD. Clinico-pathological characteristics of BRCA1- and BRCA2-related breast cancer. Research Support, Non-U.S. Gov't. *Seminars in surgical oncology*. Jun 2000;18(4):287-95.
24. National Institute for Health and Care Excellence (NICE). *Advanced breast cancer: diagnosis and treatment*. 2009. https://www.nice.org.uk/guidance/CG80/evidence
25. National Institue for Health and Care Excellence (NICE). *Early and locally advanced breast cancer: diagnosis and treatment*. Vol. 2018. 2009. https://www.nice.org.uk/guidance/CG80/evidence
26. National Institute for Health and Clinical Excellence. *National costing report: Early and locally advanced breast cancer/Advanced breast cancer*. 2009.
27. Bates T, Kearins O, Monypenny I, Lagord C, Lawrence G. Clinical outcome data for symptomatic breast cancer: the Breast Cancer Clinical Outcome Measures (BCCOM) Project. Research Support, Non-U.S. Gov't. *Br J Cancer*. Aug 4 2009;101(3):395-402. doi:10.1038/sj.bjc.6605155
28. Effects of chemotherapy and hormonal therapy for early breast cancer on recurrence and 15-year survival: an overview of the randomised trials. Meta-Analysis
Research Support, Non-U.S. Gov't. *Lancet*. May 14-20 2005;365(9472):1687-717. doi:10.1016/S0140-6736(05)66544-0
29. Heijnsdijk EA, Warner E, Gilbert FJ, et al. Differences in natural history between breast cancers in BRCA1 and BRCA2 mutation carriers and effects of MRI screening-MRISC,

© 2024 Sun L et al. *JAMA Network Open.*

MARIBS, and Canadian studies combined. Research Support, Non-U.S. Gov't. *Cancer Epidemiol Biomarkers Prev*. Sep 2012;21(9):1458-68. doi:10.1158/1055-9965.EPI-11-1196

30. Canadian Cancer Society. *Cancer information: Breast cancer*. 2023. https://cancer.ca/en/cancer-information/cancer-types/breast

31. Department of Health. *NHS reference costs 2015 to 2016*. 2016. https://www.gov.uk/government/publications/nhs-reference-costs-2015-to-2016

32. Early Breast Cancer Trialists' Collaborative Group. Effects of chemotherapy and hormonal therapy for early breast cancer on recurrence and 15-year survival: an overview of the randomised trials. *Lancet*. May 14-20 2005;365(9472):1687-717. doi:10.1016/S0140-6736(05)66544-0

33. Manchanda R, Sun L, Patel S, et al. Economic Evaluation of Population-Based BRCA1/BRCA2 Mutation Testing across Multiple Countries and Health Systems. *Cancers (Basel)*. Jul 17 2020;12(7)doi:10.3390/cancers12071929

34. Sask Cancer Agency. *Pan-Canadian Oncology Drug Review*. 2017. http://www.saskcancer.ca/health-professionals-article/pan-canadian-oncology-drug-review

35. Kozlow W, Guise TA. Breast cancer metastasis to bone: mechanisms of osteolysis and implications for therapy. Research Support, N.I.H., Extramural
Research Support, Non-U.S. Gov't
Research Support, U.S. Gov't, Non-P.H.S.
Research Support, U.S. Gov't, P.H.S.
Review. *Journal of mammary gland biology and neoplasia*. Apr 2005;10(2):169-80. doi:10.1007/s10911-005-5399-8

36. UK CR. Breast Cancer (C50), One-Year Age Standardised Net Survival by Stage, Adults (Ages 15-99 Years), England 2014. https://www.cancerresearchuk.org/health-professional/cancer-statistics/statistics-by-cancer-type/breast-cancer/survival

37. Wapnir IL, Anderson SJ, Mamounas EP, et al. Prognosis after ipsilateral breast tumor recurrence and locoregional recurrences in five National Surgical Adjuvant Breast and Bowel Project node-positive adjuvant breast cancer trials. Randomized Controlled Trial
Research Support, N.I.H., Extramural. *J Clin Oncol*. May 1 2006;24(13):2028-37. doi:10.1200/JCO.2005.04.3273

38. Anderson SJ, Wapnir I, Dignam JJ, et al. Prognosis after ipsilateral breast tumor recurrence and locoregional recurrences in patients treated by breast-conserving therapy in five National Surgical Adjuvant Breast and Bowel Project protocols of node-negative breast cancer. Clinical Trial
Randomized Controlled Trial
Research Support, N.I.H., Extramural. *J Clin Oncol*. May 20 2009;27(15):2466-73. doi:10.1200/JCO.2008.19.8424

39. Gennari A, Conte P, Rosso R, Orlandini C, Bruzzi P. Survival of metastatic breast carcinoma patients over a 20-year period: a retrospective analysis based on individual patient data from six consecutive studies. Research Support, Non-U.S. Gov't. *Cancer*. Oct 15 2005;104(8):1742-50. doi:10.1002/cncr.21359

40. Canadian Cancer Society. *Screening for breast cancer*. 2022. https://cancer.ca/en/cancer-information/cancer-types/breast/screening

41. National Institute for Health and Care Excellence. *Familial breast cancer: Classification and care of people at risk of familial breast cancer and management of breast cancer and related risks in people with a family history of breast cancer*. 2013. *NICE clinical guideline CG164 ed*.

© 2024 Sun L et al. *JAMA Network Open.*

42. Cuzick J, Sestak I, Bonanni B, et al. Selective oestrogen receptor modulators in prevention of breast cancer: an updated meta-analysis of individual participant data. *Lancet*. May 25 2013;381(9880):1827-34. doi:10.1016/S0140-6736(13)60140-3
43. Tran DT, Palfrey D, Welsh R. The Healthcare Cost Burden in Adults with High Risk for Cardiovascular Disease. *PharmacoEconomics - Open*. 2021/09/01 2021;5(3):425-435. doi:10.1007/s41669-021-00257-8
44. Who Is at Risk for Coronary Heart Disease? Accessed 17 March, 2018. https://www.nhlbi.nih.gov/health-topics/coronary-heart-disease#Risk-Factors
45. Nova Scotia Health system. *Women & Children's Health Program*. 2023. Accessed 15 January 2023. https://www.nshealth.ca/about-maternal-and-child-health-services
46. Hanly P, Timmons A, Walsh PM, Sharp L. Breast and prostate cancer productivity costs: a comparison of the human capital approach and the friction cost approach. *Value Health*. May 2012;15(3):429-36. doi:10.1016/j.jval.2011.12.012
47. National Ovarian Cancer Coalition. Ovarian Cancer Recurrence: Discussion With an Expert. Accessed 7 Nov, 2018. http://ovarian.org/component/content/article/33/385
48. Statistics Canada. *Life expectancy and other elements of the complete life table, three-year estimates, Canada, all provinces except Prince Edward Island*. 2022. https://www150.statcan.gc.ca/t1/tbl1/en/tv.action?pid=1310011401
49. Evans DG, Lalloo F, Ashcroft L, et al. Uptake of risk-reducing surgery in unaffected women at high risk of breast and ovarian cancer is risk, age, and time dependent. *Cancer Epidemiol Biomarkers Prev*. Aug 2009;18(8):2318-24.
50. Canadian Cancer Statistics Advisory Committee in collaboration with the Canadian Cancer Society, Statistics Canada and the Public Health Agency of Canada. *Canadian Cancer Statistics 2021*. Canadian Cancer Society; 2021. https://cdn.cancer.ca/-/media/files/research/cancer-statistics/2021-statistics/2021-pdf-en-final.pdf
51. Le Page C, Rahimi K, Köbel M, et al. Characteristics and outcome of the COEUR Canadian validation cohort for ovarian cancer biomarkers. *BMC Cancer*. 2018/03/27 2018;18(1):347. doi:10.1186/s12885-018-4242-8
52. Huzarski T, Byrski T, Gronwald J, et al. Ten-Year Survival in Patients With BRCA1-Negative and BRCA1-Positive Breast Cancer. *Journal of Clinical Oncology*. 2013/09/10 2013;31(26):3191-3196. doi:10.1200/JCO.2012.45.3571
53. Bordeleau L, Panchal S, Goodwin P. Prognosis of BRCA-associated breast cancer: a summary of evidence. Review. *Breast Cancer Res Treat*. Jan 2010;119(1):13-24. doi:10.1007/s10549-009-0566-z
54. Rennert G, Bisland-Naggan S, Barnett-Griness O, et al. Clinical outcomes of breast cancer in carriers of BRCA1 and BRCA2 mutations. *N Engl J Med*. Jul 12 2007;357(2):115-23.
55. McLaughlin JR, Rosen B, Moody J, et al. Long-term ovarian cancer survival associated with mutation in BRCA1 or BRCA2. *J Natl Cancer Inst*. Jan 16 2013;105(2):141-8. doi:10.1093/jnci/djs494
56. Parrish HM, Carr CA, Hall DG, King TM. Time interval from castration in premenopausal women to development of excessive coronary atherosclerosis. *Am J Obstet Gynecol*. Sep 15 1967;99(2):155-62.

© 2024 Sun L et al. *JAMA Network Open.*
