# Supplementary material for: Economic Evaluation of Population-Based BRCA1 and BRCA2 Testing in Canada
Source: JAMA Netw Open. 2024 Sep 12;7(9):e2432725. doi: 10.1001/jamanetworkopen.2024.32725 (PMC11393724; doi:10.1001/jamanetworkopen.2024.32725)
Supplement: Supplement 2. — Data Sharing Statement [file jamanetwopen-e2432725-s002.pdf]

Data Sharing Statement

Sun. Economic Evaluation of Population-Based BRCA1 and BRCA2 Testing in Canada. *JAMA Netw Open*. Published September 12, 2024. doi:10.1001/jamanetworkopen.2024.32725

Data
Data available: No

Additional Information
Explanation for why data not available: Data are largely obtained from the published literature (there is no individual patient level data in this analysis) and summary data used are already provided in the manuscript. But we are happy to consider any reasonable request for data from others. This can be done by writing to the corresponding author.
